# Antibacterial Properties and Potential Mechanism of Serum from Chinese Alligator

**DOI:** 10.3390/microorganisms10112210

**Published:** 2022-11-08

**Authors:** Meng-Yuan Hu, Yi-Wen Chen, Zhi-Fan Chai, Yin-Zhi Wang, Jian-Qing Lin, Sheng-Guo Fang

**Affiliations:** 1 MOE Key Laboratory of Biosystems Homeostasis and Protection, State Conservation Center for Gene Resources of Endangered Wildlife, College of Life Sciences, Zhejiang University, Hangzhou 310058, China; 2 Guangdong Provincial Key Laboratory of Marine Disaster Prediction and Prevention, Guangdong Provincial Key Laboratory of Marine Biotechnology, Institute of Marine Science, Shantou University, Shantou 515063, China

**Keywords:** serum, antibacterial activity, *Alligator sinensis*, protein, complement system

## Abstract

The Chinese alligator (*Alligator sinensis*) is an ancient reptile with strong immunity that lives in wetland environments. This study tested the antibacterial ability of Chinese alligator serum (CAS) against *Klebsiella pneumoniae*, *Escherichia coli*, *Staphylococcus aureus*, and *Pseudomonas aeruginosa* and analyzed the potential underlying mechanisms. Results showed that the CAS had a marked antibacterial effect on *K. pneumoniae*, *E. coli*, and *P. aeruginosa*, while *S. aureus* was only mildly affected. However, these effects disappeared when Protease K was added to the serum. The serum proteome analysis revealed that the antibacterial ability of CAS was produced by interactions among various proteins and that the complement proteins played a major antibacterial role. Therefore, we made relevant predictions about the structure and function of complement component 3. In addition, sequence alignment and phylogenetic analysis of complement component 3d (C3d) in four mammalian species and two alligator species showed that the amino acids that make up the acid pocket on the concave surface of alligator C3d are not identical to those in mammals. This study provided evidence that CAS elicits significant antibacterial effects against some pathogens and provides the basis for further development of novel antibacterial drugs.

## 1. Introduction

Since the discovery of penicillin, an increasing number of novel antibiotics have been created. Despite this fact, the number of viable antibiotics is dwindling as bacteria continue to develop resistance through factors such as penicillinase, drug-resistant plasmids, and transmissible fluoroquinolone resistance [1]. Moreover, multi-drug resistant bacteria and “superbugs” have emerged. Therefore, the development of antibacterial therapies that can be utilized as alternatives to antibiotics with less chance of resistance are urgently required. In 1933, the bactericidal effect of normal serum was first mentioned [2], and recently, there have been many studies on the potential bactericidal effect of serum. A growing number of biological agents against multi-drug resistant strains have emerged, from sources including American alligator (*Alligator mississippiensis*) [3], Komodo dragons (*Varanus komodoensis*) [4], and zebrafish (*Danio rerio*) [5].

The Chinese alligator (*Alligator sinensis*) is an endangered freshwater alligator in China that lives in wetland habitats. Interestingly, although there are abundant microorganisms in the natural habitat of Chinese alligators, they are rarely infected, which indicates that they have innate resistance to these pathogenic microorganisms. Studies have revealed that the serum of the American alligator (*Alligator mississippiensis*), which is closely related to the Chinese alligator, has a strong antibacterial ability [6]. The analysis of immune-related genes in the genome of Chinese alligator indicates that Chinese alligator has a developed innate immune system [7]. However, the details of this antimicrobial effect and the mechanism of action involved with Chinese alligator serum (CAS) have not yet been elucidated.

In this study, based on previous studies on the immune capacity of alligator serum, antimicrobial test and proteomic analysis were combined to explore the antibacterial effect and potential mechanism of Chinese alligator serum, and the immune proteins in alligator serum and mammalian serum were compared and analyzed, providing a reference for the antibacterial ability of Chinese alligator serum and laying a foundation for its antibacterial application. This is the first study on the antibacterial effect of Chinese alligator serum.

## 2. Materials and Methods

### 2.1. Reagent Preparation

Mueller–Hinton broth (MHB) and Mueller–Hinton agar (MHA) were prepared according to Coolaber (China), according to the manufacturer’s instructions. The Chinese alligator serum (CAS) was extracted from the blood of alligator tail veins with serum separation tubes, and the fetal bovine serum (FBS) and alligator serum were preserved in centrifuge tubes at −20 °C. The sample collection was performed with permission from the State Forestry Administration of China [Forest Conservation Permission Document (2014) 1545] and the Animal Ethics Committee of Zhejiang University (ZJU2015-154-13). Penicillin-Streptomycin Solution (P/S) was prepared from a mixture of 10,000 units/mL penicillin and 10,000 μg/mL streptomycin, then diluted with 1:100 medium and preserved in a centrifuge tube at −20 °C and utilized as the experimental solution. Protease K (Generay, China) was preserved in centrifuge tubes at −20 °C.

### 2.2. Bacterial Strains

We used the following ATCC registered strains maintained by Zhejiang University School of Medicine: *Staphylococcus aureus* (25923), *Klebsiella pneumoniae* (13883), *Escherichia coli* (25922), and *Pseudomonas aeruginosa* (27853).

### 2.3. Bacterial Cultures

The *S. aureus*, *K. pneumoniae*, *E. coli*, and *P. aeruginosa* were preserved at −80 °C then introduced to MHB when they needed to be activated. Each 5 μL bacteria was mixed with 1ml MHB and cultured for 6 h at 37 °C in an incubator (MMM, Germany). Then the mixed solution was streaked onto MHB plates. Following culturing at 37 °C for 10 h, the individual bacterial colonies were selected with sterilized toothpicks then inoculated into the MHB medium, and cultured at 37 °C for 12 h to the logarithmic phase, when the absorbance of the bacterial solution at 600 nm was measured with a spectrophotometer (GE, China) to reach 0.20–0.25. The concentration of the bacterial solution was 10^8^ CFU/mL. The bacterial solution was diluted 100 times and then utilized as the experimental bacterial solution.

### 2.4. Antibacterial Properties in Relation to CAS Concentration

As the Chinese alligator is an endangered species, in order to preserve the finite amount of serum, the experiment was designed as follows. Briefly, we took 96-well culture plates, zoned, and marked each plate. For each plate, 100 μL MHB was pipetted to wells 2–11 via a pipette gun (Eppendorf, Hamburg, Germany), and 200 μL MHB was pipetted into well 12. Then 200 μL P/S or serum was pipetted into well 1, and 100 μL of the respective treatment was pipetted to well 2. The liquid was mixed adequately with a pipette gun to suck it up and blow it back, and this step was repeated 3–5 times. Then 100 μL was removed from well 2 to and pipetted into well 3, this operation was repeated, until reaching well 10. The 100 μL solution in well 10 was not mixed in this manner and remained unchanged. Then, we pipetted 100 μL of the bacteria solution into wells 1–11 (Figure S5). Following culturing at 37 °C for 18 h, spectrophotometry of the solution was conducted with SynergyMx M5 Full-band Multifunctional Microplate reader (Molecular Devices, San Jose, CA, USA). This experiment was repeated three times.

### 2.5. Antibacterial Ability of the CAS

According to the experimental results of the previous experiment, we took the 96-well culture plates and pipetted the samples (Figures S6 and S7). Then we measured the spectrophotometry of each sample every two hours until the twelfth hour (*P. aeruginosa* takes longer to reach the stationary phase, so it needed 14 h while others needed 12 h).

### 2.6. Proteome Analysis of the CAS

#### 2.6.1. Protein Preparation

Samples from two Chinese alligators were lysed with protein lysate, and then PMSF with a final concentration of 1 mM and EDTA with a final concentration of 2 mM was added. DTT was added with a final concentration of 10 mM after 5 min. The supernatant was extracted following ultrasonic treatment for 15 min and centrifugation at 25,000× *g* for 20 min.

Five times the volume of precooled acetone was added to the supernatant. Following precipitation at −20 °C for 2 h and centrifugation at 16,000× *g* for 20 min, the supernatant was discarded. Next, we collected the precipitation, added protein lysate to lyse, and added PMSF with a final concentration of 1 mm and EDTA with a final concentration of 2 mm, respectively; after 5 min, DTT with a final concentration of 10 mm was added. The supernatant was extracted following ultrasonic treatment for 15 min and centrifugation at 25,000× *g* for 20 min. The supernatant was treated with DTT at a final concentration of 10 mm at 56 °C for 1 h to reduce and open the disulfide bond. The final concentration of 55 mm IAM was added, and the chamber was left for 45 min to allow for alkylation of the cysteine. Precooled acetone was added at a proper amount. Following precipitation at −20 °C for 2 h, and centrifugation at 25,000× *g* for 20 min, the supernatant was discarded. The precipitate was lysed in 200 μL 0.5M TEAB (Applied Biosystems, Foster City, CA, USA) for 15 min. Finally, centrifugation at 25,000× *g* for 20 min and then the proteins in the supernatant were extracted and kept at −80 °C for further analysis.

#### 2.6.2. TMT Protein Labeling and Mass Spectrometry Analysis

A total of 100 μg protein was taken out of each sample solution and was digested with Trypsin Gold (Promega, Madison, WI, USA) at 37 °C for 12 h, and the ratio of protein: trypsin was 20:1. Then the peptides were processed according to the manufacture’s guidelines for TMT (ThermoFisher Scientific, CA, USA), and the two samples were labeled with reagents 129 and 130, respectively.

An LC-20AB HPLC Pump system (Shimadzu, Kyoto, Japan) and a 4.6 × 250 mm Ultremex SCX column, which contains 5-μm particles (Phenomenex, Torrance, CA, USA), were used for chromatography of the sample. The mixed peptide was labeled, dried, and then reconstituted in 4 mL buffer A (25 mL NaH_2_PO_4_ in 25% CAN, pH 2.7). After being loaded onto the column in which the rate of the flow was set as 1 mL/min, the sample was eluted with a gradient: 10 min of buffer A, then 5% buffer B (25 mM NaH_2_PO_4_, 1 M KCl in 25% ACN, pH 2.7), followed by a 20-min linear gradient which raised the buffer B from 5% to 60%. Next, the proportion of buffer B was raised to 100% in 2 min and maintained for 1 min, then fell back to 5% to equilibrate with buffer A for 10 min before the next turn. The whole process was conducted with monitoring at an absorbance of 214 nm. Every 1 min the fractions were collected once. Finally, 12 fractions were treated by StrataX C18 column (Phenomenex, Torrance, CA, USA) to desalt, then were vacuum dried.

Each dried fraction was reconstituted in buffer A (5% ACN, 0.1% FA) to 0.5 μg/μL, then centrifuged for 10 min at 20,000× *g* to get rid of the undissolved matter. Five μL of each fraction (containing about 2.5 μg protein) was loaded onto a 2 cm C18 trap column at flow rate of 8 μL/min for 4 min; then the sample was carried into the analytical C18 column at the rate of 300 nL/min, eluted, and transported to the LC-20AD Nano HPLC (Japan). Then, samples wereeluted at 5% buffer B (95% ACN, 0.1%FA) for 5 min, followed by a 35-min linear gradient raising the proportion of buffer B from 5% to 35%, then 60% in 5 min, 80% in 2 min, and finally were maintained for 2 min. Finally, the proportion was set back to 5% in 1 min and equilibrated for 10 min.

The peptides were loaded into Q-Exactive (ThermoFisher Scientific, CA, USA). The primary resolution was set at 70,000, and the secondary resolution was set at 17,500. Fifteen parent ions with charges of 2+~5+ and peak intensity over 20,000 were sorted out for secondary analysis. High-energy collision dissociation (HCD) operating mode with a normalized collision energy setting of 27 (±12%) was used for selecting peptides that were detected in orbitrap. Dynamic Exclusion duration was set for 15 s. The electrospray voltage was 1.6 kV. Automatic gain control (AGC) was performed by orbitrap. For full MS, the AGC target was 3e6, and for MS2, it was 1e5. As for MS scans, 350 to 2000 Da was set as the *m*/*z* scan range. For MS2, the range was 100–1800.

#### 2.6.3. Bioinformatics Analysis

Raw data files were converted to MGF files using Proteome. Discoverer 1.2 (PD 1.2, Thermo) [5600 msconverter] and the MGF files were searched. Protein identification was performed by using Mascot search engine (Matrix Science, London, UK; version 2.3.02) (Table 1). Pathway annotations of the proteins were conducted against the KEGG database.

### 2.7. Analysis of the Complement Component 3 Found in the CAS

Based on the analysis results in Section 2.6, the complement proteins in the serum of the Chinese alligator were considered to play an important role in the process of antimicrobial activity, and C3 was found to be an important protein for initiation of alternative pathways in the complement system. Therefore, we analyzed the structure and function of C3.

The primary structure of the C3 in CAS was obtained from UniProt, and the amino acid sequence was translated from nucleic acid sequencing rather than measured experimentally according to UniProt Protparam and then used to analyze both sequence characteristics and sequence stability.

The secondary structure of C3 was predicted and analyzed with PredictProtein [8]. The function annotation of PredictProtein was used to test subcellular localization and related GO terms, to determine information about the function of C3 in the immune system.

The tertiary structure of C3 was predicted and analyzed by Phyre2 [9], and the template used for the predicted structure was PDB ID: 2b39. The tertiary structure image of C3 was edited and labeled with PyMol.

### 2.8. Sequence Alignment and Phylogenetic Analysis of Complement Component 3d

We downloaded the C3d protein amino acid and nucleotide sequence of human (*Homo sapiens*), mouse (*Mus musculus*), cow (*Bos taurus*), pig (*Sus scrofa*), Chinese alligator (*Alligator sinensis*), and American alligator (*Alligator mississippiensis*) from National Center for Biotechnology Information (NCBI). ClustalW2 [10] was used to perform multiple sequence alignment of amino acid sequences of C3d proteins from six species, and the alignment results were visualized using ENDscript [11].

Subsequently, the nucleotide and amino acid sequences of the six species were used for phylogenetic analysis. The phylogenetic trees were constructed via the neighbor-joining (NJ) method with 1000 bootstrap replicates using MEGA software [12].

## 3. Results

### 3.1. Effect of Concentration of CAS on the Antibacterial Activity

**Figure 1:**
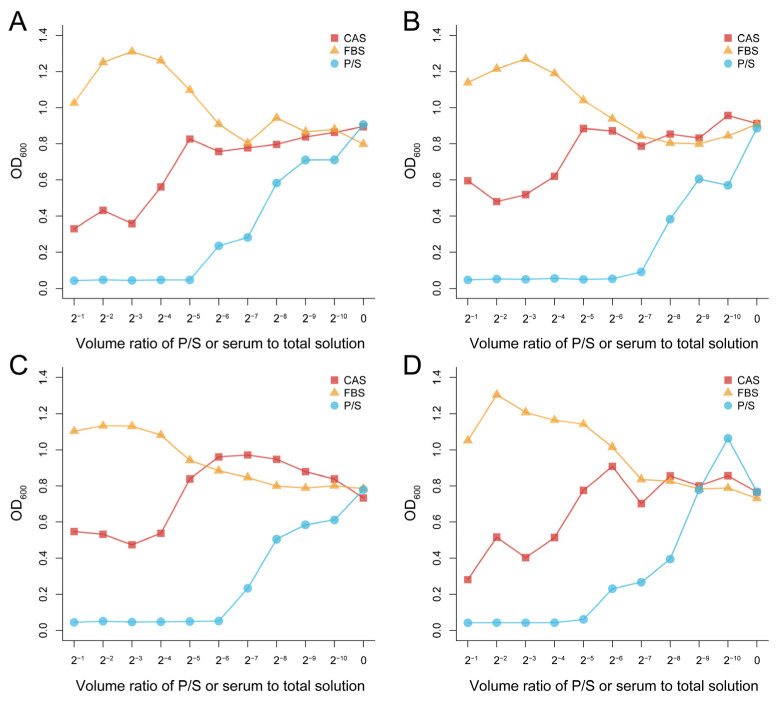
Antibacterial effect of Chinese alligator serum against *Klebsiella pneumoniae* (**A**), *Staphylococcus aureus* (**B**), *Escherichia coli* (**C**), and *Pseudomonas aeruginosa* (**D**) at different concentrations. There is 100 μL of the bacteria solution and 100 μL of the serum or P/S in well 1. In wells 2 to 10, the concentration of serum or P/S was reduced to 1/2 of the previous well consecutively. No serum or P/S was added to well 11, only the bacterial solution and culture medium. All of the bacterial solutions in the plates had reached the platform stage. CAS: Chinese alligator serum, FBS: fetal bovine serum, P/S: Penicillin-Streptomycin Solution.

Compared with fetal bovine serum (FBS) and Penicillin-Streptomycin Solution (P/S), the antibacterial effect was generally enhanced with the increase of CAS concentration. When the volume ratio of serum to solution was more than 1/16, the growth of four of the bacteria in this experiment was inhibited to varying degrees. However, when the volume ratio was less than 1/32, the growth of the four bacteria was not inhibited, and instead was promoted, which was similar to the effect of fetal bovine serum (Figure 1).

### 3.2. Antibacterial Effect of the CAS on Different Strains at a Given Concentration

**Figure 2:**
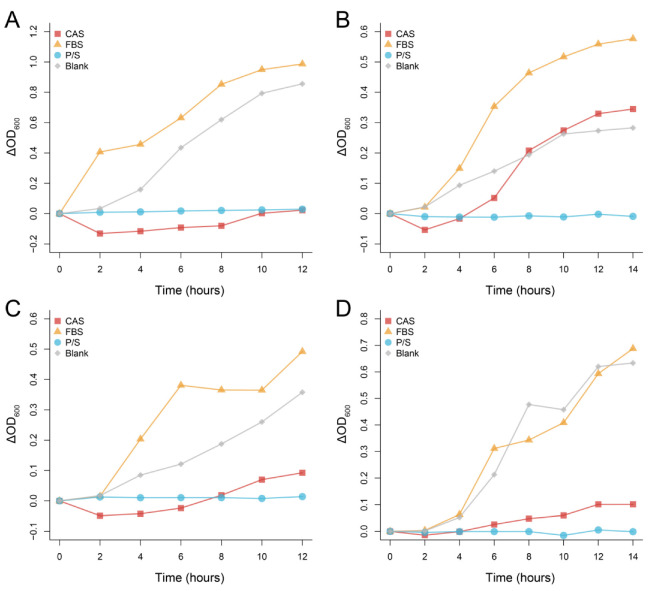
Antibacterial effect of Chinese alligator serum on *Klebsiella pneumoniae* (**A**), *Staphylococcus aureus* (**B**), *Escherichia coli* (**C**), and *Pseudomonas aeruginosa* (**D**) at a given concentration. Changes in absorbance were obtained by comparing the absorbance of each sample at different times with the initial absorbance. CAS: Chinese alligator serum, FBS: fetal bovine serum, P/S: Penicillin-Streptomycin Solution.

At the given concentration, the CAS produced a significant antimicrobial effect against *K. pneumoniae*, *E. coli*, and *P. aeruginosa*; however, little benefit was conferred to *S. aureus* (Figure 2). In the first 2 h, the absorbance of the bacterial solution in the presence of the CAS decreased significantly, which may suggest that some consumable antimicrobial substances are present in the serum.

### 3.3. Antibacterial Effect of the CAS with Protease K

**Figure 3:**
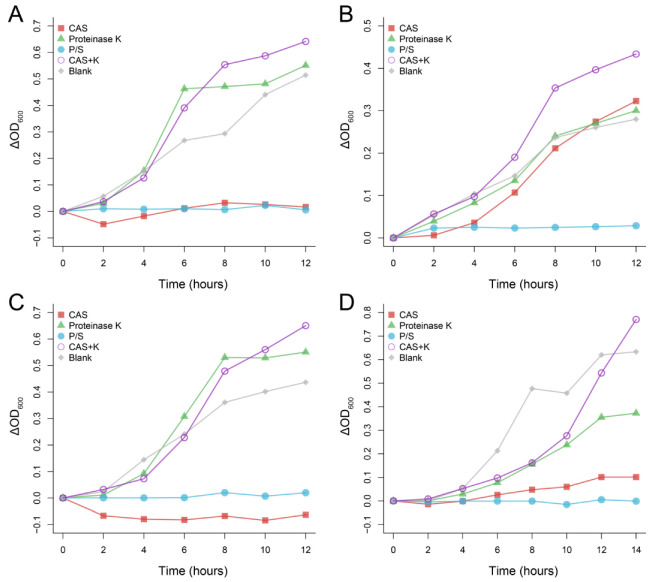
Antibacterial effect of Chinese alligator serum after proteinase K treatment on *Klebsiella pneumoniae* (**A**), *Staphylococcus aureus* (**B**), *Escherichia coli* (**C**) and *Pseudomonas aeruginosa* (**D**). Changes in absorbance were obtained by comparing the absorbance of each sample at different times with the initial absorbance. CAS: Chinese alligator serum, P/S: Penicillin-Streptomycin Solution, K: proteinase K.

After Protease K was added to CAS, its antibacterial effect on the four bacteria disappeared, and Protease K itself neither promoted nor inhibited the four bacteria (Figure 3), which indicated that the main antibacterial component in CAS was protein.

### 3.4. Proteomic Analysis of the CAS

We then used iTRAQ technology to analyze the proteins in the serum of Chinese alligator, and identified a total of 595 proteins and 3337 peptides, including 3251 unique peptides.

According to the results of the KEGG pathway enrichment, there were two main types of pathways: Pathogen invasion and defense pathways, such as Leishmaniasis pathway, *Staphylococcus aureus* infection pathway, amoebiasis pathway, viral myocarditis pathway, complement and coagulation cascades pathway, and immunodeficient disease pathways, such as systemic lupus erythematosus pathway, primary immunodeficiency pathway, and rheumatoid arthritis pathway (Table S2).

In the serum proteome, complement protein and antibody protein were the main proteins involved in these pathways. In addition, the antimicrobial peptide, perforin, and avidin were also involved. We summarized the relationship between these proteins and the four bacteria used in the experiments (Table 1).

According to previous studies, antimicrobial peptides and avidin inhibited or were bound to four different kinds of bacteria used in this experiment [13,14,15,16,17]. Perforin has only been associated with the inhibition of *S. aureus* [18,19]. Complement proteins inhibited *K. pneumoniae*, *E. coli*, and *P. aeruginosa* [20,21,22]. *S. aureus* might have escaped detection by the immune system by lysing the complement C3 [23,24]. This information, in conjunction with our results, suggests that the complement protein was most likely to play a significant role in the antibacterial process, and C3 was found to be the key protein involved.

The complement system activated alternative pathways in the serum, produced membrane attack complexes, degraded bacterial cell membranes, and played an important antimicrobial role in conjunction with other proteins, such as antimicrobial peptides and the perforins, which also participated in the antimicrobial process [25]. In addition, a variety of antibody proteins in the serum participated in the activation of the complement membrane attack complex and enhanced the antibacterial effect [26]. Avidin, when combined with four kinds of bacteria in the experiment promoted the antibacterial function of antimicrobial peptides [27]. In addition to immune-related proteins, there were a large number of signal transduction proteins in the serum proteome, which were also involved in the antimicrobial activity. This indicated that the antimicrobial effect of crocodile serum was the result of the combined action of various antimicrobial mechanisms.

### 3.5. Analysis of Complement C3 Protein in Chinese Alligator

According to the analysis of ProtParam, the C3 protein contains 1655 amino acids with a relative molecular weight of 186,168.84 and a theoretical isoelectric point of 6.34. Alanine was the primary amino acid (Table S3). The C3 protein possesses 204 negative residues, 193 positive residues, and the molecular formula is C_8331_H_13189_N_2195_O_2487_S_71_. According to the stability test results, the instability coefficient was 39.03 < 40, and the protein structure was stable.

According to the prediction results, the secondary structure of Chinese alligator C3 protein was dominated by random coils. In addition, GO prediction results of C3 protein of Chinese alligator showed that C3 protein was involved in the binding process of various substances including protein and metal ions, and it was speculated that C3 protein of Chinese alligator might be involved in a variety of regulatory functions, such as regulating kinase activity (Figures S2 and S3).

The tertiary structure of the Chinese alligator complement C3 protein was predicted using the protein with SMTL ID 2b39.2 (mammalian C3) as a template, and it was displayed using PyMol (Figure S4).

### 3.6. Sequence Analysis and Phylogenetic Analysis of Complement Component 3d

C3d is one of the final hydrolysis products of C3 protein [28] and is an important molecular adjuvant, which can link non-specific immunity with specific immunity [29]. The crystal structure of C3d contains both convex and concave surfaces, with the convex surface mediating covalent binding to the target antigen and the concave surface containing the acidic pocket that binds to B cell complement receptor 2 (CR2).

**Figure 4:**
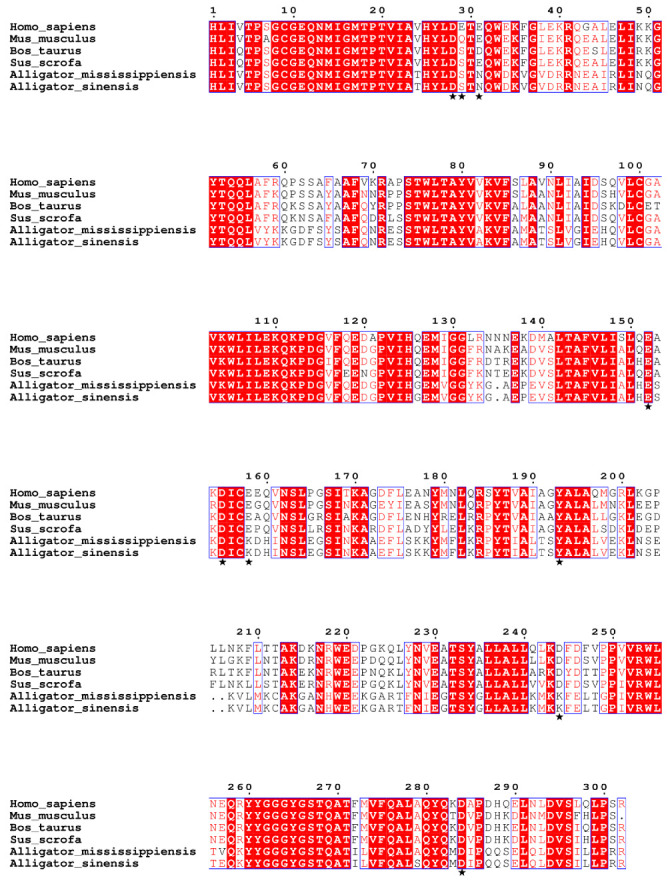
Multiple sequence alignment of C3ds from two alligators and four mammals. Amino acid residues that bind to B cell complement receptor 2 are marked with asterisks.

Multiple sequence alignment of C3d amino acid sequences from multiple species using ClustalW (Figure 4). In mammalian C3d, the four amino acids Cyc9, Glu11, Gln12, and His125 covalently bind to the C3d convex antigen to aid in antigen presentation [30,31]. Sequence alignment showed that these four amino acid sites were strictly conserved in the six species, suggesting that alligators may have the structural basis for covalently binding ligands.

We further compared the amino acid sequences in the C3d acidic pocket of the six species. Asp28, Glu152, Asp155, Tyr193, and Asp284 in the acidic pocket are conserved in six species, and other sites in the acidic pocket are mutated (Table 2). The acidic amino acid residues Glu29, Glu31, Glu158, and Asp244 in mammals are mutated to neutral Ser29 and Asn31 and basic Lys158 and Lys244, respectively, in alligators. Mutations of these acidic amino acids to neutral and basic amino acids suggest that the acidic pocket of mammalian C3d differs from that of alligators.

**Figure 5:**
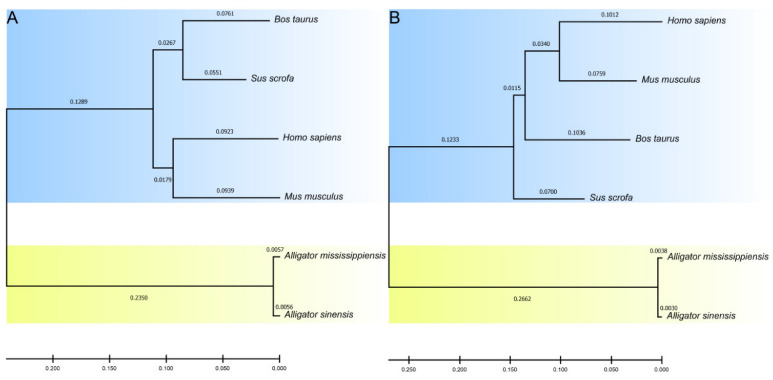
Phylogenetic relationships of C3d nucleotide sequences (**A**) and amino acid sequences (**B**) in two alligators and four mammals.

To understand the evolutionary relationship between alligator and mammalian C3d, we performed phylogenetic analysis of their nucleotide and amino acid sequences (Figure 5). In the evolutionary tree of nucleotide sequence and amino acid sequence, alligators and mammals were divided into two distinct clusters, suggesting that the C3ds of alligators have been significantly differentiated from that of mammals, but there is no obvious differentiation between the two kinds of alligators.

## 4. Discussion

The antibacterial activity of serum has been studied in a variety of crocodilians [6,32,33], but there is still a lack of relevant research in Chinese alligators. In this study, we tested the antibacterial activity of Chinese alligator serum as an antibacterial agent against four common pathogenic bacteria and analyzed the potential antibacterial mechanisms. In addition, we performed a sequence alignment of the alligator and mammalian C3d proteins to analyze the differences in the complement system of the two types of animals. In conclusion, this study hopes to provide a reference for the development and utilization of the antibacterial ability of Chinese alligator serum.

The results showed that CAS produced a marked antibacterial effect against *K. pneumoniae*, *E. coli*, and *P. aeruginosa*, and mildly affected *S. aureus*, prior to treatment with Protease K. The four bacterial strains used in this study were not drug-resistant strains, so as to preliminarily test the antibacterial ability of Chinese alligator serum. In future studies, we will use multi-drug resistant strains to test the antimicrobial ability of Chinese alligator serum against drug-resistant strains. Although some studies have shown the presence of antibacterial peptides in alligator serum [3], according to the enrichment results of the KEGG pathway, complement protein was found to be the most active in eliciting antibacterial activity. However, the complement recognition mechanism and broad-spectrum antibacterial properties imparted to the pathogens still require further exploration. Complement proteins and antibody proteins have been widely found in various organisms. Previous research has reported that the diversity of C3 proteins in the complement pathway was conducive to the recognition of various microorganisms [34]. Researchers found two genes that code for isoforms of the complement C3 protein with quite different sequences in crocodilian genomes, while birds and mammals express only a single isoform [35]. Furthermore, the functional activity of the C3 protein was almost undetectable in the fetal bovine serum, while other complement components were detected at lower levels [36]. Moreover, different kinds of antibodies were found in animal serum from unrelated species. Therefore, the species differences found in the C3 protein and antibody protein might explain the broad-spectrum antibacterial effect of the CAS.

The rupture of the membrane of alligator serum-treated cells can be observed under electron microscopy, which is consistent with the mode of action of membrane attack complex, perforin, and antimicrobial peptide [33]. In 2005, Mark Merchant et al. found that temperature and EDTA influenced the antibacterial effect of the Mississippi alligator serum, and they suggested that the main antibacterial substance was complement protein rather than the antimicrobial peptides [6]. This information supports the experimental results found in the current study.

*K. pneumoniae*, *E. coli*, *P. aeruginosa*, and *S. aureus* were widely distributed in the living environment of the Chinese alligator [37,38,39,40]. *S. aureus* is Gram-positive, while the other three bacteria are Gram-negative. Previous research has reported that the membrane attack complex produced by the complement system could directly lyse the Gram-negative bacteria, while the Gram-positive bacteria were not lysed due to the thick peptidoglycan cell wall [24], and some Gram-positive bacteria, represented by *S. aureus*, could secrete a variety of complement inhibitors, which prevented the complement from acting. In an experiment to test the antibacterial ability of 23 different crocodile serum against eight Gram-negative bacteria, the bacteria were all inhibited by different kinds of crocodile serum, but no single crocodile serum could inhibit all eight bacteria at the same time [41]. Thus, different kinds of crocodile serum produced different antibacterial effects against different strains, and Gram-positive bacteria might possess stronger resistance to crocodile serum; however, the mechanism of action requires further study.

Complement protein C3 is the most abundant complement component in human serum, serves as the intersection of the classical, alternative and lectin pathways of complement activation, and is a special molecule that occupies a central position in host defense mechanisms [30]. Under the action of C3 convertase, C3 is cleaved into two fragments, C3a and C3b, which play important roles in both the classical activation pathway and the alternative activation pathway of complement [28]. Subsequently, C3b can be hydrolyzed into iC3b and C3dg fragments and finally degraded to C3d. As the final hydrolysis product of C3b, C3d can bind to B cell complement receptor 2 (CR2) through the acidic pocket and is an important link between innate and adaptive immunity [29]. The amino acid sequence alignment results showed that the amino acids constituting the acidic pocket are partially conserved between mammals and alligators. Negatively charged Glu29, Glu31, Glu158, and Asp244 in human C3d were mutated to neutral Ser29 and Asn31 and positively charged Lys158 and Lys244, respectively, in Chinese alligator. This suggests that an acidic pocket is formed on the concave surface of the alligator C3d protein, distinct from that of mammalian C3d proteins. This acidic pocket also binds CR2, but does so in different ways, which may indicate differences in the immune system between mammals and alligators. In addition, phylogenetic analysis revealed that the amino acid sequence and nucleotide sequence of C3d were highly conserved in their respective groups in mammals and alligators, respectively. Especially in alligators, the amino acid sequence of C3d of Chinese alligator and American alligator is only different at two sites. These results suggest that alligator C3d has a different sequence and structure from mammals, forming its own unique acidic pocket for binding to CR2.

## 5. Conclusions

In this study, we used the combination of antibacterial testing and serum proteomic analysis to explore the antibacterial ability of Chinese alligator serum. The results showed that complement proteins played a key role in this ability, but the mechanism of pathogen recognition and broad-spectrum bacteriostasis still needs to be further explored. In addition, serums from different species of crocodile have different antibacterial effects on different bacterial species, and Gram-positive bacteria may have stronger resistance to crocodile serum, and the mechanism of which needs to be further studied.

## Figures and Tables

**Table 1:** Relationship between the proteins and bacteria.

	Cathelicidin	Perforin	Complement	Avidin
*K. pneumoniae*	Inhibition	Uncertain	Inhibition	Binding (inhibition effect is negligible)
*S. aureus*	Inhibition	Inhibition	Not inhibition	
*E. coli*	Inhibition	Uncertain	Inhibition	
*P. aeruginosa*	Inhibition	Uncertain	Inhibition	

**Table 2:** Comparison of the acidic pocket amino acids between humans and alligators.

Human	Alligators
Asp28	Asp
**Glu29**	**Ser**
**Glu31**	**Asn**
Glu152	Glu
Asp155	Asp
**Glu158**	**Lys**
Tyr193	Tyr
**Asp244**	**Lys**
Asp284	Asp

The bold indicates that the amino acids of human and alligator are inconsistent at this site.

## Data Availability

The Chinese alligator reference genome is available from GenBank (assembly accession: GCA_000455745.1). The TMT data generated in this work have been deposited in the ProteomeXchange with identifier PXD017090.

## Supplementary Materials

The following supporting information can be downloaded at: https://www.mdpi.com/article/10.3390/microorganisms10112210/s1, Figure S1: Secondary structure composition of complement C3 protein in Chinese alligator; Figure S2: Molecular functional ontology of Chinese alligator complement C3 protein; Figure S3: Biological process ontology of Chinese alligator complement C3 protein; Figure S4: Predicted tertiary structure of Chinese alligator complement C3 protein; Figure S5: Sample configuration scheme for the determination of serum inhibitory concentration of Chinese alligator in 96-well plate; Figure S6: Sample configuration scheme of 96-well plate for Chinese alligator serum antibacterial experiment; Figure S7: Sample configuration scheme for determination of antibacterial components in Chinese alligator serum in 96-well plate; Table S1: Parameters used in proteins identification; Table S2: The top 20 pathways of serum protein annotated results in the KEGG database; Table S3: Composition of amino acid residues in complement C3 protein of Chinese alligator, human and cattle.
