# Supplementary material for: Antibacterial Properties and Potential Mechanism of Serum from Chinese Alligator
Source: Microorganisms. 2022 Nov 8;10(11):2210. doi: 10.3390/microorganisms10112210 (PMC9698150; doi:10.3390/microorganisms10112210)
Supplement: Supplementary file 1 [file microorganisms-10-02210-s001.zip › microorganisms-1921364-supplementary.pdf]

Supplementary Figures

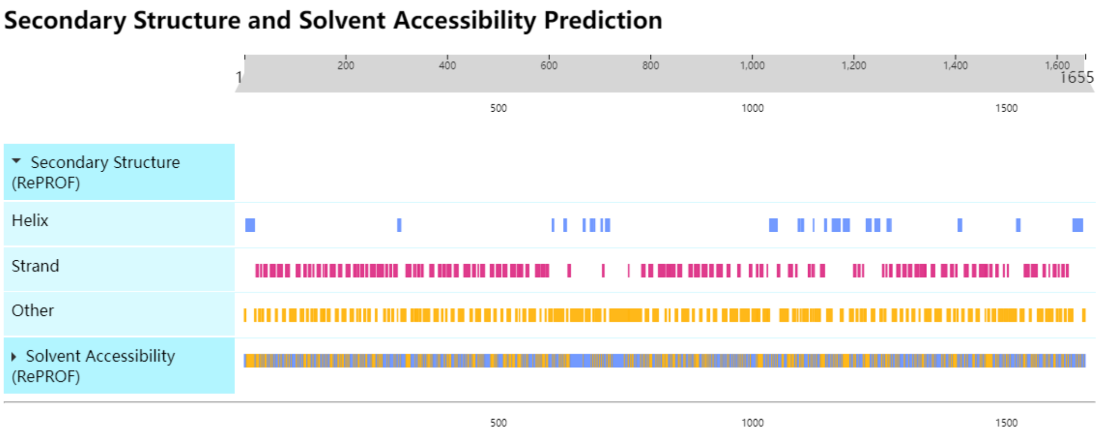

Figure S1. Secondary structure composition of complement C3 protein in Chinese alligator.

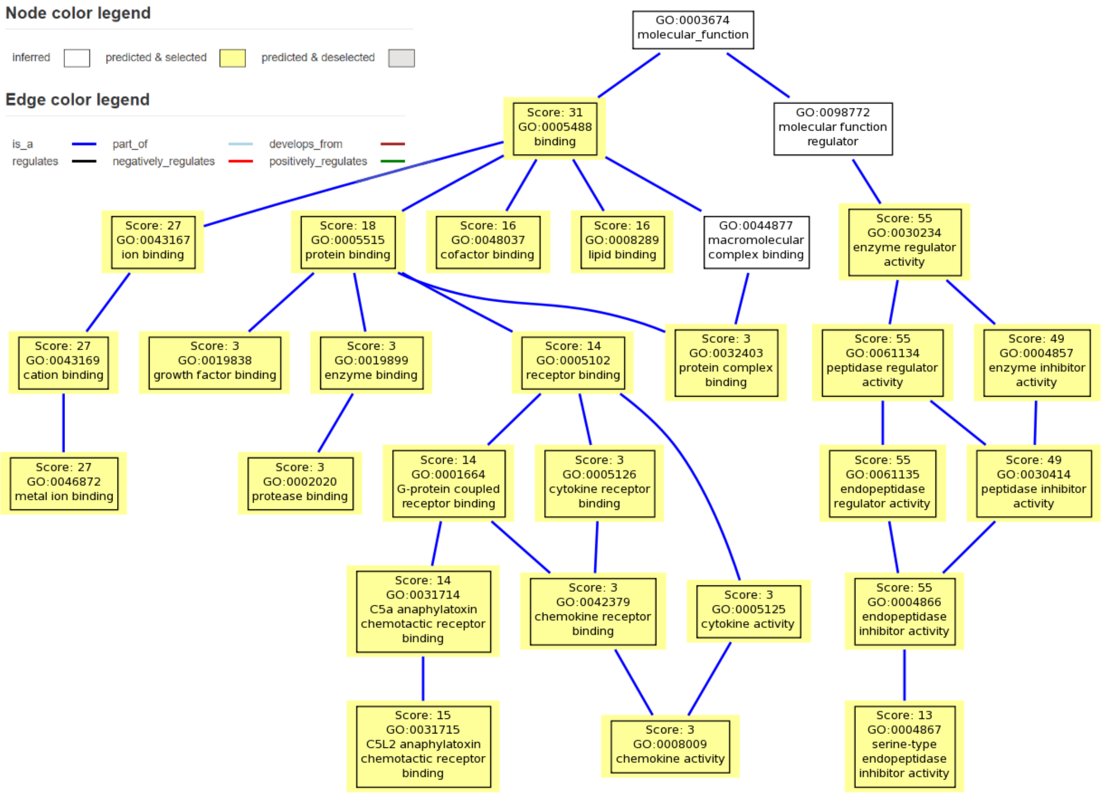

Figure S2. Molecular functional ontology of Chinese alligator complement C3 protein.

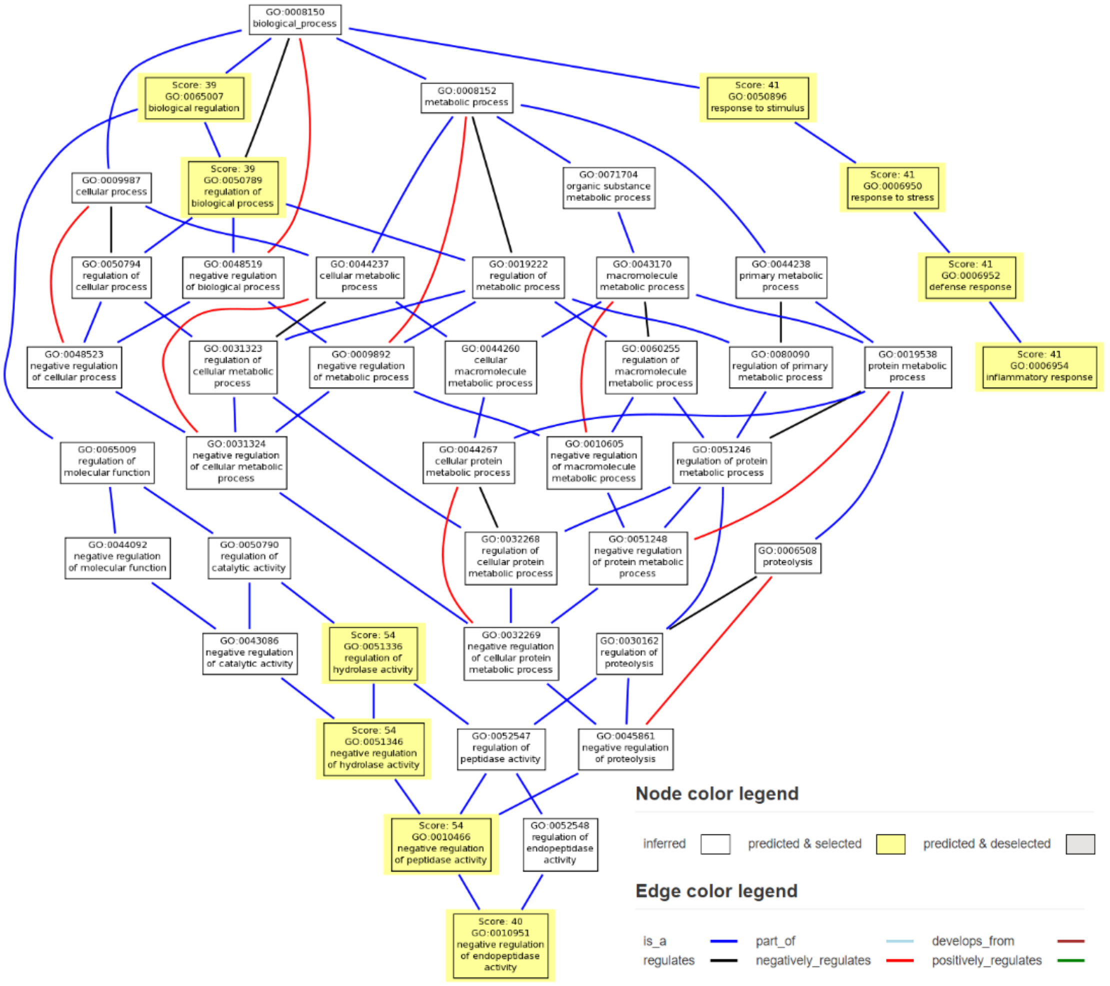

Figure S3. Biological process ontology of Chinese alligator complement C3 protein.

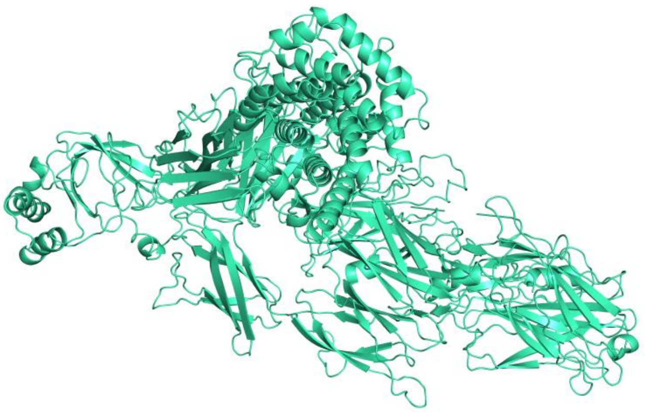

Figure S4. Predicted tertiary structure of Chinese alligator complement C3 protein.

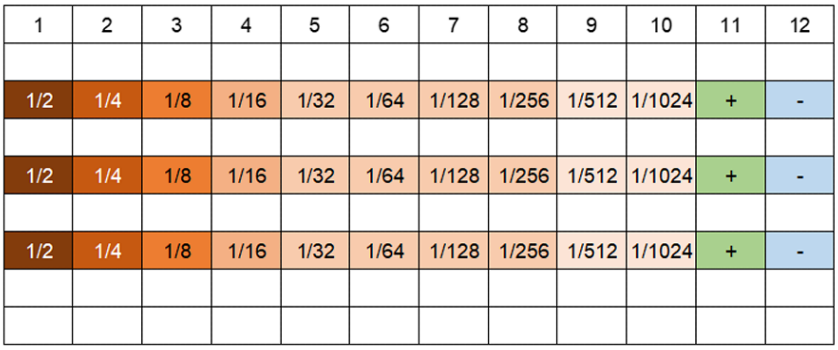

Figure S5. Sample configuration scheme for the determination of serum inhibitory concentration of Chinese alligator in 96-well plate.

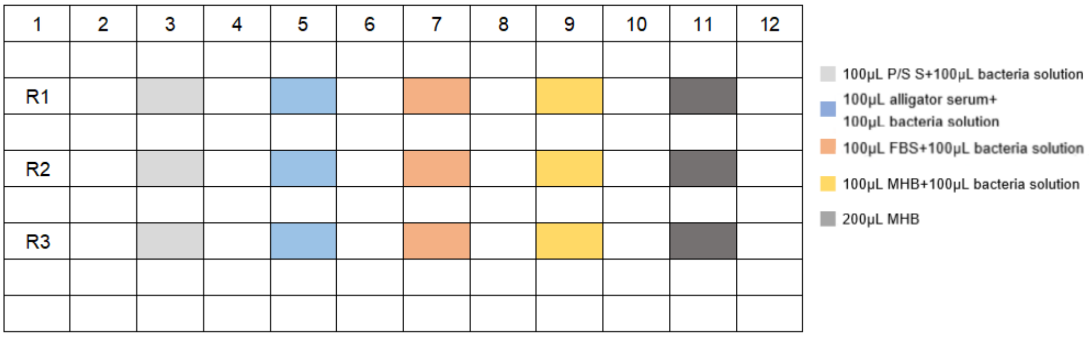

Figure S6. Sample configuration scheme of 96-well plate for Chinese alligator serum antibacterial experiment.

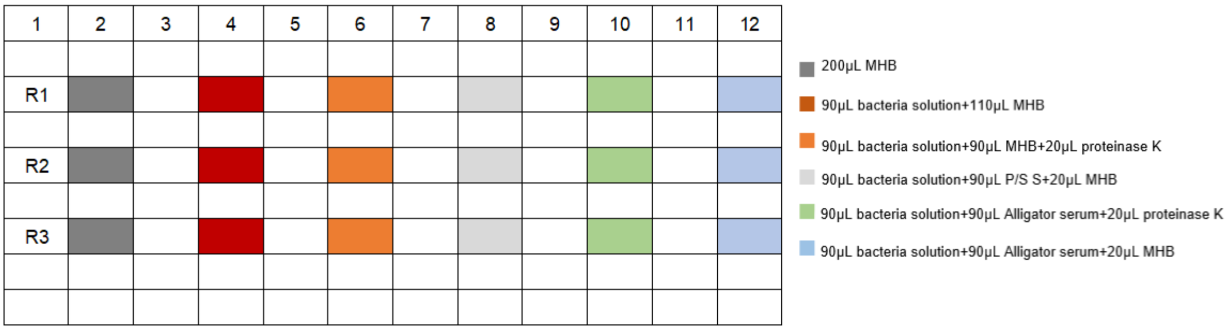

Figure S7. Sample configuration scheme for determination of antibacterial components in Chinese alligator serum in 96-well plate.

Supplementary Tables

Table S1. Parameters used in proteins identification.

Item	Value
Type of search	MS/MS Ion search
Enzyme	Trypsin/P
Fragment Mass Tolerance	± 0.02 Da
Mass Values	Monoisotopic
Variable modifications	Oxidation (M)
Peptide Mass Tolerance	10 ppm
Instrument type	Default
Max Missed Cleavages	2
Fixed modifications	Carbamidomethyl(C), TMT6plex (N-term)， TMT6plex (K)
Database	uniprot

Table S2. The top 20 pathways of serum protein annotated results in the KEGG database.

**KEGG Pathway**	**Proteins with pathway annotation (488)**	**Pathway ID**
Phagosome	90 (18.44%)	ko04145
PI3K-Akt signaling pathway	85 (17.42%)	ko04151
**Leishmaniasis**	**82 (16.8%)**	**ko05140**
Dilated cardiomyopathy	80 (16.39%)	ko05414
**Staphylococcus aureus infection**	**79 (16.19%)**	**ko05150**
**Amoebiasis**	**70 (14.34%)**	**ko05146**
**Viral myocarditis**	**67 (13.73%)**	**ko05416**
Hematopoietic cell lineage	67 (13.73%)	ko04640

Tuberculosis	66 (13.52%)	ko05152
**Systemic lupus erythematosus**	**65 (13.32%)**	**ko05322**
Natural killer cell mediated cytotoxicity	64 (13.11%)	ko04650
**Primary immunodeficiency**	**64 (13.11%)**	**ko05340**
**Complement and coagulation cascades**	**64 (13.11%)**	**ko04610**
**Rheumatoid arthritis**	**59 (12.09%)**	**ko05323**
Epstein-Barr virus infection	58 (11.89%)	ko05169
Cell adhesion molecules (CAMs)	57 (11.68%)	ko04514
Regulation of actin cytoskeleton	54 (11.07%)	ko04810
Rap1 signaling pathway	53 (10.86%)	ko04015
Pertussis	52 (10.66%)	ko05133
Calcium signaling pathway	50 (10.25%)	ko04020

Table S3. Composition of amino acid residues in complement C3 protein of Chinese alligator, human and cattle.

	**Chinese alligator**		**Human**		**Cattle**	
	**Quantity**	**Proportion**	**Quantity**	**Proportion**	**Quantity**	**Proportion**
**Ala (A)**	87	5.30%	96	5.90%	95	5.80%
**Arg (R)**	63	3.90%	82	5.00%	81	4.90%
**Asn (N)**	68	4.20%	61	3.70%	64	3.90%
**Asp (D)**	84	5.10%	90	5.50%	102	6.20%
**Cys (C)**	29	1.80%	27	1.60%	27	1.60%
**Gln (Q)**	73	4.50%	90	5.50%	88	5.40%
**Glu (E)**	119	7.30%	123	7.50%	112	6.80%
**Gly (G)**	96	5.90%	95	5.80%	96	5.90%

**His (H)**	30	1.80%	28	1.70%	32	2.00%
**Ile (I)**	103	6.30%	83	5.10%	96	5.90%
**Leu (L)**	137	8.40%	150	9.10%	144	8.80%
**Lys (K)**	130	8.00%	113	6.90%	122	7.40%
**Met (M)**	39	2.40%	35	2.10%	30	1.80%
**Phe (F)**	62	3.80%	60	3.70%	54	3.30%
**Pro (P)**	70	4.30%	80	4.90%	74	4.50%
**Ser (S)**	112	6.90%	106	6.50%	95	5.80%
**Thr (T)**	107	6.60%	103	6.30%	105	6.40%
**Trp (W)**	18	1.10%	17	1.00%	17	1.00%
**Tyr (Y)**	60	3.70%	57	3.50%	62	3.80%
**Val (V)**	146	8.90%	145	8.80%	143	8.70%
**Pyl (O)**	0	0.00%	0	0.00%	0	0.00%
**Sec (U)**	0	0.00%	0	0.00%	0	0.00%
